# Metalloproteinase-2 in failed back surgery syndrome caused by epidural fibrosis: can it play a role in persistent pain?

**DOI:** 10.3389/fnhum.2023.1248943

**Published:** 2023-09-19

**Authors:** Joanna Bielewicz, Beata Daniluk, Piotr Kamieniak

**Affiliations:** ^1^Department of Neurology, Medical University of Lublin, Lublin, Poland; ^2^Institute of Psychology, Marie Curie-Skłodowska University in Lublin, Lublin, Poland; ^3^Department of Neurosurgery, Medical University of Lublin, Lublin, Poland

**Keywords:** failed back surgery syndrome, disk herniation, metalloproteinase-2, tissue inhibitor of metalloproteinase-2, microdiscectomy

## Abstract

**Purpose:**

Failed Back Surgery Syndrome (FBSS) occurs in 10–40% of patients treated surgically due to disk herniation (DH). There are several factors that can cause a predisposition to FBSS, but the exact pathomechanism has not been elucidated. The aim of this study was to investigate Metalloproteinase-2 (MMP-2) and Tissue Inhibitor of Metalloproteinase-2 (TIMP-2) activities in a homogeneous group of FBSS patients with epidural fibrosis in comparison to its activity in patients with surgically treated DH.

**Methods:**

DH, FBSS, and control (CG) groups consisted of 30 subjects. The patients were assessed clinically by the Numerical Rating Scale (NRS), McGill Pain Questionnaire (SF -MPQ), Oswestry Disability Index (ODI), and Beck Depression Inventory (BDI). Serum concentrations of MMP-2 and TIMP-2 were measured by using the immunoenzymatic method.

**Results:**

There was a significantly higher MMP-2 expression (medians: 4797.49 vs. 2656.65; *p* < 0.0001) and TIMP-2 concentration (medians: 166.40 vs. 109.60; *p* < 0.0001) in the DH compared to the CG. Significantly higher MMP-2 expression (4219.95 vs. 2656.65; *p* < 0.0001) and TIMP-2 concentration (medians: 150.17 vs. 109.60; *p* = 0.0003) were also found in the FBSS compared to the CG. The activity of MMP-2, measured as MMP-2/TIMP-2, did not significantly change between the DH, FBSS, and CG. MMP2 expression (*p* < 0.0001) and TIMP-2 concentration (*p* < 0.0001) were significantly higher in the DH than FBSS.

**Conclusion:**

Results indicate the presence of a contribution of MMP-2 and TIMP-2 in DH and FBSS. Unchanged activity of MMP-2 can indicate an insufficiency in the MMP-2 repair system in both diseases. Lower MMP-2 expression and TIMP-2 concentration in the FBSS group can reflect the chronicity of the process.

## 1. Introduction

According to the International Association for the Study of Pain (IASP), Failed Back Surgery Syndrome (FBSS) is defined as “Lumbar spinal pain of unknown origin either persisting despite surgical intervention or appearing after surgical intervention for spinal pain originally in the same topographical location” (Baber and Erdek, 2016). FBSS occurs in 10–40% of patients treated surgically due to lumbar pain who show resistance to conservative treatment. Half of the patients diagnosed with FBSS had undergone more than one spinal surgery (Thomson, 2013). The broad definition of FBSS does not take into account the etiological variety of preoperative disorders. Nonetheless, FBSS fulfills the criteria of neuropathic pain (NP) (Treede et al., 2008). There are several factors identified that can cause a predisposition to FBSS, such as preoperative psychiatric disorders, obesity, smoking, inaccurate diagnosis, improper choice of the type of surgery, intraoperatively inadequate decompression, postoperative recurrent disc herniation (DH), progression of degenerative changes in adjacent segments, altered biomechanics of vertebral column, “battered” nerve syndrome, the presence of foraminal stenosis, and nerve root irritation or nerve roots entrapment in epidural fibrosis (Sebaaly et al., 2018). In the majority of patients, DH proceeded by the intervertebral disc degeneration (IVDD) process is a self-limiting disorder that is effectively treated pharmacologically or resolves spontaneously (Komori et al.,1996; Haro, 2014). The latter phenomenon depends on an efficient system of many interplaying substances, from cytokines, which are engaged in neuroinflammation, the initial phase of DH, to metalloproteinases (MMPs) (Doita et al., 2001). MMPs seem to be important elements in this chain reaction directly responsible for the dissolution of detrimental fragments of DH, which can favorably affect the healing process (Goupille et al., 1998; Grang et al., 2001). However, the surgical intervention is considered in 10% of patients with DH (Haro, 2014). In this group, the above, self-limiting processes are not effective, resulting in persistent pain. This can indicate the insufficiency of the MMPs system. MMPs are a family of zinc-dependent endoproteases engaged in multiple processes of tissue remodeling by degrading various proteins of the extracellular matrix (ECM) (Cui et al., 2017). Their activity is regulated by many factors including endogenous tissue inhibitors of metalloproteinases (TIMPs) of a crucial role. Increased MMP expression/activity or decreased TIMPs could lead to an MMP/TIMP imbalance and results in various pathological conditions (Raeeszadeh-Sarmazdeh et al., 2020). MMP-2 belongs to a subgroup of gelatinases which cooperates with collagenases in degradation of collagen I, II, and III. They are involved in such processes as tissue repair, inflammation, and remodeling of ECM during DH.

Several investigations have indicated MMP-2 as one of the key factors involved in pain pathogenesis (Kawasaki et al., 2008; Liou et al., 2013; Miranpuri et al., 2017). Decrease in MMP-2 expression leads to an alleviation of NP and an improvement in functional recovery after spinal cord injury in animal models (Miranpuri et al., 2017). The aim of this study was to investigate MMP-2 activity in FBSS patients in comparison to its activity in DH patients who qualified for surgery. The results could contribute to an explanation of the probable pathomechanism of FBSS.

## 2. Materials and methods

### 2.1. Patients

In total, 107 patients admitted to the Department of Neurosurgery of the Medical University of Lublin were prospectively enrolled in the study. All patients received written and verbal information regarding the study design and procedures. Signed consent was obtained from all participating patients. The study protocol and consent forms were approved by the Ethics Committee of the Medical University of Lublin in Poland in accordance with binding legislation (approval number KE-0254/148/2020).

A total of 107 patients were separated into two groups. The first group, made up of 33 individuals (19 female-F, 14 male-M), was diagnosed as an FBSS group according to the IASP definition [1]. The inclusion criteria for the FBSS group were as follows: (1) previous surgery due to DH; (2) chronic NP, which was classified as refractory after at least 2 years of pharmacological treatment, meaning a lack of a minimum 50% relief in pain intensity (PI) according to Numeric Rating Scale (NRS); (3) the presence of epidural fibrosis magnetic resonance imaging (MRI) confirmed after the procedure; epidural fibrosis was diagnosed using 1.5 T MRI with T1- and T2- weighted sagittal, horizontal, and axial sequences with a 4-mm section thickness and with gadolinium (Gd). It was seen as hypo- or isointense on T1-weighted and hyperintense on T2-weighted images change with enhancement after Gd. The amount of epidural fibrosis was graded on a scale of 1–4 for each quadrant of each imaging slice encompassing the operative level as follows: 1 was assigned if the quadrant area showed no or minimal fibrosis, i.e., < 25% of the quadrant was filled with fibrosis, 2 if it showed ≥25% and < 50% fibrosis, 3 if ≥50% and < 75%, and 4 if ≥75%.

Based on MRI results, 18 patients were presented with Grade 3 and 15 patients with Grade 4 according to the Ross grading system (Ross et al., 1999).

Exclusion criteria were as follows: (1) the results of a psychological assessment indicating a psychiatric disorder and (2) current ongoing corticosteroids treatment.

The second group of 74 patients (32 F, 42 M) with subacute sciatica pain due to lumbar DH were qualified for microdiscectomy (DH group). The inclusion criteria for microdiscectomy were as follows: (1) the age of patients was between 18 and 80 years; (2) a diagnosis of clinically symptomatic DH; (3) the confirmation of clinical diagnosis by MRI. The exclusion criteria were as follows: (1) previous corticosteroid therapy for 3 months preceding surgery; (2) the presence of previous spine surgery or spinal stenosis; (3) co-existence of other medical conditions such as rheumatoid diseases, diabetes, cancer, psychiatric diseases, recent surgery for another reason than DH, pregnancy, alcohol or drug abuse, or other disorders with clinical pain presentation.

The control group (CG) consisted of 30 subjects recruited from patients with chronic varicose veins. The mean age and sex groups corresponded with the DH and FBSS groups. Patients from the CG group did not complain about pain having neuropathic features. They did not use analgesic drugs.

The demographic, clinical, and radiological characteristics of patients are given in Table 1.

**Table 1:** Characteristic of patients in assessed groups.

Data	DH group	FBSS group	Control group
Sex	18 females 12 males	18 females 12 males	18 females 12 males
Age (years) (median and range)	53 (48–55)	55 (51–60)	54.5 (47–58)
Occupation 0 1	9 (30%) 21 (70%)	9 (30%) 21 (70%)	9 (30%) 21 (70%)
Diagnosis	Disc Herniation	Failed Back Surgery Syndrome	Chronic varicose veins
Mechanism of injury	3-root compression L3/L4 10-root compression L4/L5 17-root compression L5/S1	FBSS caused by epidural fibrosis	No injury of spinal roots
Modic scale			
	1 patient – 0 15 patients- 1 14 patients - 2	15 patients – 2 15 patients - 3	N/A
Pfiermann scale	1 patient - 1 1 patient - 2 14 patient - 3 14 patient - 4	14 patients – 3 16 patients - 4	N/A
Comorbidity	5- hypertension arterialis mild 1-asthma bronchialis 1-chronic gastritis	4-hypertension arterialis mild 1-tachycardia 2-chronic gastritis	30-varicose veins 2-hypertension arterialis mild
Medications			No analgesic drugs
NSAIDs	28 subjects (average time 4 months)	28 (average time 80 monts)	None
Opioids	27 (average time 6.5 months)	25 (average time 120 months)	None
Others analgesic	28 (average time 5 months)	27 (average time 8.5 months)	None
Physiotherapy	30 subjects (average time 3 month)	30 subjects (average time 6 months)	None

DH, disc herniation; FBSS, failed back surgery syndrome; NSAID, non-steroidal anti-inflammatory drugs; Occupation 0, mainly intellectual work; 1, mainly physical work.

Finally, DH, FBSS, and CG patients were matched according to sex, age, and occupation. Every group consists of 30 patients: 18 females and 12 males. There was no FBSS in the DH group for the 5 years of observation. The median age was 53 (48–55) in DH, 55 (51–60) in FBSS, and 54.5 years (47–58) in CG. There were no statistical significant differences between groups in terms of sex (*p* = 1,00), age (*p* = 0,3,259), and occupation (*p* = 1,00).

### 2.2. Neurosurgical procedure

All patients of the FBSS group underwent several procedures in the past, with the average being 3 (min = 1, max = 10). All patients from DH were operated on by one surgeon who used the standard microdiscectomy method. The procedure was carried out under general anesthesia.

### 2.3. Clinical assessment

A total of 33 patients from FBSS and 74 from DH were assessed according to NRS in the back (NRS B) and in the leg (NRS L) separately using the Pain Rating Index (PRI) and Present Pain Intensity (PPI) as part of the McGill Pain Questionnaire (SF -MPQ), Oswestry Disability Index (ODI), and Beck Depression Inventory (BDI).

### 2.4. Biochemical assessment

Blood samples were collected from all 33 patients of the FBSS, 74 patients of the DH, and 30 patients of the CG groups.

Venous blood (5 mL) was drawn in the morning on the day of surgery, before discectomy, and immediately centrifuged at 4000 g for 15 min. The serum samples were promptly frozen at −80 C for further analysis.

*MMP-2 assessment.* MMP-2 expression was determined by gelatin zymography based on visualization of free-gelatine areas digested by MMP-2, serum samples were diluted 1 to 50 with redistilled water, and mixed in 1 to 4 ratios with a sample buffer containing 10% sodium dodecyl sulfate (SDS). The enzyme was separated by polyacrylamide gel electrophoresis (PAGE) on 10% gel with 0.05% gelatine type A from porcine skin (G2500) (Sigma–Aldrich, United States). After separation gels were washed for 1 h in order to remove SDS. Gel incubation was carried out overnight at 37\u00B0C degree in the buffer containing 1% Triton X-100, pH 7.2. Gels were stained with 0.1% Coomassie Blue R-250 in 20% methanol and 10% acetic acid, and subsequently the stain was removed in 20% methanol and 10% acetic acid solution. MMP-2 was detected as clear bands on a blue background. The enzymes were identified by comparing their localization with molecular mass standards (SM0441) (Fermentas Life Sciences, Germany) as well as with the standards of gelatinase (R&D Systems Inc., United States). Zymographic gels were scanned and quantified with Image J software (National Institute of Health, United States). The expression of MMP-2 was shown as the optical density (OD) of the substrate lysis zone.

*TIMP-2 assessment.* The plasma concentrations of TIMP-2 were determined by the immunoenzymatic method using commercially Human TIMP-2 Quantikine ELISA Kit (R&D Systems Inc., United States) according to the manufacturer’s instructions. The concentrations are expressed in ng/ml for TIMPs.

*MMP-2 activity*. It is measured as a ratio of MMP-2 to TIMP-2 concentration.

### 2.5. Statistical analysis

Statistical analysis of the data was performed with the use of Statistica software (ver. 13 PL) and MedCalc (ver. 20.027). Categorized variables were presented as absolute numbers and percentages. The chi-square test was used to assess the differences in the distribution of categorized variables. Due to the non-normal distribution of continuous variables (assessed by Shapiro–Wilk test), medians and corresponding interquartile ranges were used in order to present data concentration and dispersion, respectively. Continuous data showed non-normal distribution in comparisons of two independent groups using the non-parametric U-Mann–Whitney test, whereas in comparisons of more than two independent groups the ANOVA Kruskal-Wallis test was used. The *post hoc* analysis was Bonferroni corrected, and the adjusted value of *p* was set to *p*-adj < 0.05 in Kruskal−Wallis tests. The correlation coefficient of rho- Spearman’s was employed to assess the associations between variables. Since the study groups were recruited in various clinical trials, it was decided to use the propensity score matching (PSM) method to balance them in terms of basic demographic and social characteristics (sex, age, and occupation). Before PSM, the DH, FSBB and CG groups consisted of 74, 33, and 30 subjects, respectively. During PSM, the following parameters were set: sex (exact match), age (5-year difference allowed), and occupation (exact match). After PSM, all groups consisted of 30 subjects. In all analyses, the two-tailed tests were used. All results with a value of *p* below 0.05 were considered to be statistically significant.

## 3. Results

### 3.1. Comparison of clinical variables in DH and FBSS groups

Significantly longer duration of pain (medians: 120 vs. 8 months; *p* < 0.0001) and higher scores of NRS B (medians: 8 vs. 5; *p* < 0.0001), NRS L (8 vs. 5; *p* = 0.0006), PRI (medians: 17.5 vs. 13; *p* = 0.0008), PPI (3 vs. 2; *p* = 0.0210), ODI (32 vs. 22.5; *p* = 0.0017), and BECK (15 vs. 8; *p* = 0.0034) scales were found in FBSS group (Table 2).

**Table 2:** Comparison of clinical variables in DH and FBSS groups.

Variable	DH median [interquartile range]	FBSS median [interquartile range]	*p*
Duration of pain in leg [months]	8 [4–14]	120 [60–132]	<0.0001*
NRS B	5 [1–6.5]	8 [7–8]	<0.0001*
NRS L	5 [3.5–7]	8 [6–8]	0.0006*
PRI	13 [6–20.2]	17.5 [14–25]	0.0008*
PPI	2 [2–3]	3 [3–3]	0.0210*
ODI	22.5 [18–32]	32 [28–35]	0.0017*
BECK	8 [4–15]	15 [13–17]	0.0034*

*Statistically significant result.

DH, disc herniation; FBSS, failed back surgery syndrome; NRS B, numerical rating scale back; NRS L, numerical rating scale leg; PRI, pain rating index of McGill pain questionnaire (SF -MPQ); PPI, present pain intensity scale of McGill pain questionnaire (SF-MPQ); ODI, oswestry disability index; BDI, beck depression inventory.

### 3.2. Comparison of the MMP-2, TIMP-2, and MMP-2/TIMP-2 ratio in DH, FBSS, and CG

There was a significantly higher expression of MMP-2 (medians: 4797.49 vs. 2656.65; *p* < 0.0001) and concentration of TIMP-2 (medians: 166.40 vs. 109.60; *p* < 0.0001) in the DH group compared to the CG. No significant difference between the activity of MMP-2, measured as MMP-2/TIMP-2 in the DH group, compared to the CG was noticed. Also, significantly higher expression of MMP-2 (4219.95 vs. 2656.65; *p* < 0.0001) and TIMP-2 concentration (medians: 150.17 vs. 109.60; *p* = 0.0003) were found in the FBSS group compared to the CG. MMP-2/TIMP-2 was not significantly changed between FBSS and CG. The expression of MMP2 (*p* < 0.0001) and concentration of TIMP-2 (*p* < 0.0001) were significantly higher in the DH than in FBSS (Table 3).

**Table 3:** Comparison of the MMP2, TIMP2, and MMP2/TIMP2 ratio in DH, FBSS, and control groups.

Variable	DH median [interquartile range]	FBSS median [interquartile range]	Control median [interquartile range]	DH vs. FBSS *p*	DH vs. K *p*	FBSS vs. K *p*
MMP2	4797.49 [4005.28–6375.88]	4219.95 [3191.52–5567.56]	2656.65 [1892.70–2711.08]	0.1136	<0.0001*	<0.0001*
TIMP2	166.40 [148.57–199.19]	150.17 [124.55–159.05]	109.60 [71.05–127.95]	0.0162*	<0.0001*	0.0003*
MMP2/TIMP2	27.69 [22.88–36.87]	31.46 [21.56–37.27]	27.66 [21.19–34.86]	0.5748	0.5108	0.2167

*Statistically significant result.

DH, disc herniation; FBSS, failed back surgery syndrome; MMP-2, metalloproteinase-2; TIMP-2, tissue inhibitor of metalloproteinase-2.

### 3.3. Correlations of MMP-2, TIMP-2, and MMP-2/TIMP-2 with the scales of clinical assessment (NRS, SF-MPQ, ODI, and BDI) in the FBSS and DH groups

Significant changes were observed only in the DH group. A moderate negative correlation between the expression of MMP-2 and the result of the NRS-B (*rho* = −0.42; *p* = 0.0249) as well as between the concentration of TIMP-2 and the score of Beck scale for assessment of depressive signs (*rho* = −0.58; *p* = 0.0023) were noticed (Table 4).

**Table 4:** Correlations of MMP-2, TIMP-2, and MMP-2/TIMP-2 with the scales of clinical assessment (NRS, SF-MPQ, ODI, and BDI) in the FBSS and DH groups.

Variable	MMP2		TIMP-2		MMP-2/TIMP-2	
	*rho*	*p*	*rho*	*p*	*rho*	*p*
	DH					
Duration of pain in leg [months]	−0.24	0.2040	−0.34	0.0856	−0.09	0.6421
NRS B	−0.42^*^	0.0249	0.20	0.3372	−0.33	0.1133
NRS L	0.01	0.9420	−0.23	0.2753	0.16	0.4341
PRI	0.08	0.6742	−0.16	0.4256	0.13	0.5357
PPI	−0.05	0.8183	−0.06	0.7730	0.003	0.9877
ODI	0.06	0.7469	−0.25	0.2051	0.09	0.6459
BECK	−0.17	0.3903	−0.58^*^	0.0023	−0.13	0.5377
	FBSS					
Duration of pain in leg [months]	0.06	0.7651	0.03	0.8680	−0.05	0.7991
NRS B	−0.17	0.4155	−0.12	0.5503	0.01	0.9549
NRS L	−0.15	0.4353	−0.21	0.2610	0.09	0.6467
PRI	0.19	0.3055	−0.12	0.5130	0.16	0.3971
PPI	0.001	0.9946	−0.16	0.4409	0.01	0.9713
ODI	−0.05	0.7836	−0.01	0.9771	−0.01	0.9832
BECK	0.06	0.7679	0.21	0.2746	−0.05	0.8024

*Statistically significant result.

DH-disc herniation; FBSS, failed back surgery syndrome; NRS B, numerical rating scale back; NRS L, numerical rating scale leg, PRI, pain rating index of McGill pain questionnaire (SF -MPQ); PPI, present pain intensity; ODI, oswestry disability index; BDI, beck depression inventory; MMP-2, metalloproteinase-2; TIMP-2, tissue inhibitor of metalloproteinase-2.

## 4. Discussion

FBSS is heterogenic entity, diagnosed in patients with no satisfactory results after preceding DH surgeries. As mentioned in the introduction, different factors may contribute to FBSS in a particular patient. In these conditions, elucidation of the common patomechanism of FBSS is impossible. However, our investigation was performed in a homogeneous group of FBSS patients with MRIs confirming epidural fibrosis, which we consider as a valuable aspect of this study. A high prevalence of epidural fibrosis has been reported in FBSS patients in former research (Bosscher and Heavner, 2010; Lin et al., 2019). It occurs as a conglomerate of fibrotic tissue, surrounding lateral nerve roots and contributing to nerve root entrapment (Sebaaly et al., 2018; Yang et al., 2022).

An elevated expression of MMP-2 and concentration of TIMP-2 but not activity of MMP-2 were observed in the DH group in our study. These results are partially consistent with former research, indicating the important role of MMP-2 and TIMP-2 in IVDD and DH (Bachmeier et al., 2009). MMP-2 is responsible for degradation of denatured collagens and collagen IV (Ricard-Blum, 2011). MMP-2 in the extruded canine disc material showed mild upregulation over the whole course of the disease (Karli et al., 2014). Kozaci et al., 2006 analyzed the collagen and proteoglycan contents in the discs of human subjects. Pro-MMP-2, but no TIMP-2, levels were higher at early stages of the IVDD. Similarly, MMP-2 expression increased but TIMP-2 decreased, which suggests this contribution in the development of cervical DH (Zhuang et al., 2016). Pro-MMP-2 levels negatively correlated with the collagen content in herniated disc material. Crean JK et al. found increased levels of MMP-2 in discs of patients who underwent discectomy (Crean et al., 1997). Based on our results, observed MMP-2 expression upregulation in the DH group can suggest its engagement in herniated disc remodeling as an attempt of the repair process. Interestingly, we found that the profile of MMP-2, TIMP-2, and MMP-2/TIMP-2 in the FBSS is similar to the DH before operation. FBBS patients have elevated expression of MMP-2 and TIMP-2 concentration but no MMP-2 activity. This observation has pointed out the lack of efficacy of previous surgical interventions. This explanation should consider incorrect qualifications, inappropriate methods, or a delayed time of procedure. The long-lasting MMPs dysregulation cannot be normalized even when the detrimental factor (DH) is removed. In this particular group, the increased expression of MMP-2 and concentration of TIM-2 can still be maintained, probably due to the existence of epidural fibrosis as a causative factor of root compression and as a self-spinning process in the presence of persistent neuroinflammation. Pro-inflammatory cytokines promote secretion of MMPs (Cui et al., 2017). The surgery is targeted to amelioration of the pain but does not inhibit the degenerative process of IVDD still progressing (Le Maitre et al., 2007). We also cannot exclude that FBSS patients are genetically predisposed to express particular variants of MMPs. Several genes are suspected to be involved in DH with most of them encoding extracellular matrix proteins (Hirose et al., 2008). The genetic variability of MMPs can affect the recovery of patients with lumbar back pain and lumbar radicular pain (Bjorland et al., 2017). The results of our study showed an increased expression of MMP-2 and concentration of TIMP-2 in DH compared with its levels in FBSS patients, which can be related to the early phase of process of disc degeneration. On the other hand, decreased expression of MMP-2 and concentration of TIMP-2 in FBSS groups can reflect the chronicity of the process. However, in both diseases, the activity of MMP-2 remains unchanged, which can indicate the insufficiency of the MMP-2 repair system.

MMP-2 is involved in the phenomena of NP pathogenesis (Kawasaki et al., 2008; Dev et al., 2010). In animal models, after spinal nerve ligation, MMP-2 shows a delayed upregulation in injured DRG (dorsal roots ganglion) satellite cells and spinal astrocytes in a late phase of NP (Haro, 2014). Although the observations based on animals are not fully consistent with the results of research on humans (Kamieniak et al., 2019), one can assume the contribution of MMP-2 in NP. We noticed some correlations between MMP-2, TIMP-2, and MMP-2/TIMP-2 and PI in the DH group. Higher PI assessed by every applied scale of pain measurement was found in the FBSS group compared to the DH group. Worse functional status as evaluated by the ODI scale and more increased depressive signs as evaluated by BDI were also found in the FBSS group. Such results can relate to the chronicity of the process, lack of effectiveness of treatment in previous surgeries, and having had numerous surgeries. However, there was no correlation between the clinical assessment scores and the expression of MMP-2 or concentration of TIMP-2 and MMP-2/TIMP-2 in the FBSS group. Only the increased expression of MMP-2 showed a negative correlation to the PI in the back in the DH group. These findings can indicate MMP-2 contributes to the pain patomechanism in a group of patients with predominant back but not leg pain. The longer observation of stratified groups of patients, for example a group with predominant leg pain versus back pain, is mandatory.

FBSS is a heterogenic entity that requires more detailed stratification to differentiate particular subsets of patients based on the underlying patomechanism (Gatzinsky et al., 2019). Such accurate taxonomy of FBSS allowed us to determine and administer effective and targeted treatment.

## 5. Conclusion

Based on the results of our study, MMP-2 and TIMP-2 upregulation is common observation in patients of DH and FBSS caused by the detrimental presence of epidural fibrosis. Unchanged MMP-2 activity can indicate insufficiency of the MMP-2 repair system in both diseases. MMP-2 and TIMP-2 disturbances can be associated with the development of epidural fibrosis and can contribute to FBSS. The question remains direct regulation of MMP-2 will result in the healing processes and pain amelioration.

## Data availability statement

The raw data supporting the conclusions of this article will be made available by the authors, without undue reservation.

## Ethics statement

The studies involving humans were approved by the Ethics Committee of the Medical University of Lublin in Poland, in accordance with binding legislation (approval number KE-0254/148/2020). The studies were conducted in accordance with the local legislation and institutional requirements. The participants provided their written informed consent to participate in this study.

## Author contributions

JB, BD, and PK performed the material preparation, data collection, and analysis. JB wrote the first draft of the manuscript. All authors contributed to the study conception, design, read and approved the final manuscript, and commented on previous versions of the manuscript.
